# P-1531. Two against One: Ceftazidime-Avibactam plus Aztreonam vs Cefiderocol in the Treatment of Metallo-Beta-Lactamase-Producing Bacterial Infections

**DOI:** 10.1093/ofid/ofae631.1699

**Published:** 2025-01-29

**Authors:** Spencer A Harris, Vanthida Huang

**Affiliations:** Midwestern University College of Pharmacy - Glendale Campus, Peoria, Arizona; Midwestern University, College of Pharmacy-Glendale Campus, Glendale, Arizona

## Abstract

**Background:**

Metallo-beta-lactamase-producing Gram-negative bacteria (MBL-GNB) has become a global healthcare threat. MBL-GNB are resistant to all first-line antimicrobials, leading to high mortality rates. Ceftazidime-avibactam plus aztreonam (CAZ-AVI+AZT) and cefiderocol (CFDC) are treatment options due to their *in vitro* activity against MBL-GNB. However, the optimal treatment for MBL-GNB infections remains unclear. Therefore, we sought to evaluate the outcomes of CAZ-AVI+AZT vs CFDC in patients with MBL-GNB infections.

**Methods:**

Adult patients admitted to HonorHealth Network (Phoenix, AZ) from January 2018 to December 2024 who received ≥1 dose of either CAZ-AVI+AZT or CFDC with culture-confirmed MBL-GNB were included. Patients who were treated for < 48 h were excluded. The primary outcome was all-cause in-hospital mortality. Secondary outcomes were 14-day microbiological cure, post-diagnosis intensive care unit (ICU) and hospital length of stay (LOS), and duration of therapy.

**Results:**

A total of 24 patients were included, of whom 8 received CAZ-AVI+AZT and 16 received CFDC. Baseline characteristics were similar in both arms; however, CAZ-AVI+AZT patients experienced an increase in ICU admission and sepsis at baseline. Sources of infections included urine (50%), blood (42%), and respiratory tract (42%). For CAZ-AVI+AZT vs CFDC patients, all-cause in-hospital mortality occurred in 2 vs 3 patients (25% vs 19%; p = 0.72); microbiological cure occurred in 7 vs 7 patients (88% vs 44%, p = 0.11), respectively. Hospital length of stay was 9.5 ± 4 days for CAZ-AVI+AZT vs 6.5 ± 3 days for CFDC (p = 0.09). Duration of therapy and post-diagnosis ICU length of stay were similar in both groups.

**Conclusion:**

CAZ-AVI+AZT compared to CFDC demonstrated no mortality benefit; however, it may achieve greater microbiological cure. Further studies are warranted to corroborate these findings.

**Disclosures:**

**All Authors**: No reported disclosures

